# A Diagnostic Enigma of Central Versus Nephrogenic Diabetes Insipidus: What Does the Value of Copeptin Signify?

**DOI:** 10.7759/cureus.22633

**Published:** 2022-02-26

**Authors:** Dhan B Shrestha, Ranjit B Jasaraj, Roman Dhital, Esho Georges, Andriy Havrylyan

**Affiliations:** 1 Department of Medicine, Mount Sinai Hospital, Chicago, USA; 2 Department of Internal Medicine, Mount Sinai Hospital, Chicago, USA; 3 Medicine, Institute of Medicine, Kathmandu, NPL; 4 Department of Nephrology, Mount Sinai Hospital, Chicago, USA; 5 Department of Endocrinology, Mount Sinai Hospital, Chicago, USA

**Keywords:** diuresis, polyuria, polydipsia, copeptin, nephrogenic di

## Abstract

Diabetes insipidus (DI) is a rare disease characterized by hypotonic urine output and polydipsia. We report the case of a 50-year-old male admitted for diabetic ketoacidosis (DKA) in an intensive care unit with sepsis and alcoholic pancreatitis who later developed DI. He had a high copeptin level of >21.4 pmol/L, suggesting DI of nephrogenic origin. At presentation, he had DKA-associated osmotic diuresis. His later finding of elevated copeptin suggesting partial nephrogenic DI created a diagnostic enigma.

## Introduction

Diabetes insipidus (DI) is a rare disease that affects 1/25,000 people [1]. It is a part of the polyuria polydipsia syndrome, which is defined by a hypotonic urine output of >50 mL/kg body weight per 24 hours and polydipsia of >3 L/day [2]. Polyuria polydipsia syndrome can be differentiated into either primary (central or nephrogenic DI) or secondary (primary polydipsia). Central DI is caused by insufficient arginine vasopressin (AVP) synthesis or secretion in response to osmotic stimulation. On the other hand, nephrogenic DI is marked by a reduction in AVP sensitivity in the kidneys. Either central or nephrogenic DI can appear as complete or partial forms, making it challenging to differentiate between them. Primary polydipsia is characterized by excessive fluid intake and consecutive polyuria despite adequate AVP secretion and renal response. Distinguishing between the different types of DI can be challenging. It is done either by a water deprivation test or hypertonic saline stimulation together with copeptin (or AVP) measurement [3].

We report the case of a 50-year-old man with a medical history of type 2 diabetes mellitus and daily alcohol consumption who presented to the Emergency Department. He was treated for diabetic ketoacidosis (DKA) with septic shock and alcoholic pancreatitis. From the fifth day of admission, he started having polyuria with urine output of more than 6 L/day along with hypernatremia (with sodium level in 150s (mg/dL)] suggestive of DI. Hence, copeptin level was ordered which revealed a high copeptin level (>21.4 pmol/L). We report this case due to the diagnostic enigma due to high copeptin level (nephrogenic DI) and partial desmopressin responsiveness with background polyuria secondary to DKA.

## Case presentation

A 50-year-old man with a medical history of type 2 diabetes mellitus and daily alcohol consumption was brought to the Emergency Department by Emergency Medical Service (EMS) for weakness and altered mental status. As per the EMS report, he had not moved or eaten for two days. His medical history was limited as he was confused. He denied any pain or falls or any other specific complaints. Additionally, he was not sure when his last drink was.

On admission, he had a blood pressure of 72/54 mmHg, temperature of 96.8°F, pulse of 83 beats per minute, respiratory rate of 26 breaths per minute, and saturation of 96% on room air. He was oriented to person only but not to time and place. He was weak but did not have any functional neurological deficit with the muscular power of 4/5 in his both upper and lower extremities. Laboratory results showed leukocytosis with white blood cell (WBC) count of 36,000/mL, hemoglobin count 12.8 gm/dL, sodium (Na^+^) level 115 mEq/L (corrected Na-128), potassium (K^+^) level 5.2 mEq/L, chloride (Cl^-^) level 78 mEq/L, bicarbonate level <10 mEq/L, creatinine level 3 mg/dL, blood glucose level 903 mg/dL, normal anion gap, lipase >3,000 U/L, beta-hydroxybutyrate level >4.5 mmol/L, and serum osmolality 335 mOsm/kg. Urine analysis was positive for leukocyte esterase and ketone bodies. He had a high-sensitivity cardiac troponin T-test of 34 ng/L. Electrocardiogram did not show any ischemic changes. The urine drug screen was negative for opioids, cannabinoids, and benzodiazepines. Urine and blood cultures were sent.

With a working diagnosis of DKA with sepsis and alcoholic pancreatitis, he was resuscitated with fluid and treated for DKA with insulin per institutional guidelines. He was started on broad-spectrum antibiotics and admitted to the intensive care unit (ICU).

On day two of admission, he was intubated due to acute hypoxemic respiratory failure. He developed septic shock and was started on vasopressors. His blood and urine culture both grew methicillin-resistant *Staphylococcus aureus* (MRSA). After ICU treatment, his troponin and lipase trended down, but he started developing polyuria, and his sodium levels increased.

On day five, his urine output was 6.5 L, and sodium was 155 mEq/L. His serum osmolality was 335 mOsmol/Kg, urine osmolality was 165 mOsmol/Kg, with urine sodium 47 mEq/L, urine potassium 7 mEq/L, and urine chloride 47 mEq/L. His copeptin level was 40 pmol/L. He was started on 1 µg of intravenous (IV) desmopressin every 24 hours which was increased to 2 µg twice daily on the next day. He was also found to have infective endocarditis, and CT scan findings were suggestive of septic emboli to the right parietal and temporal lobe of the brain and lungs during this period. Subsequently, the patient was managed conservatively, as per the recommendations from cardiothoracic surgery.

**Figure 1:**
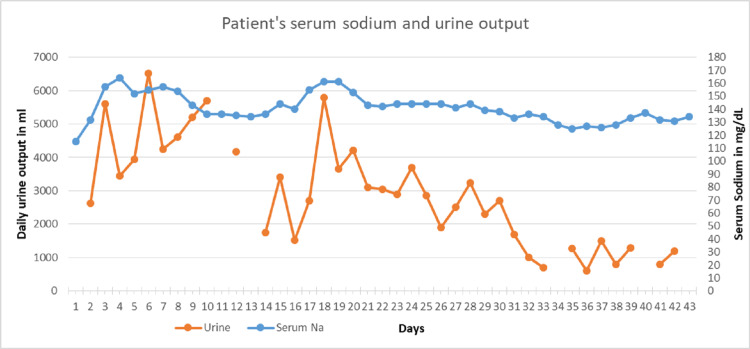
Patient’s serum sodium and urine output trend.

On day 13, his sodium was 136 mEq/L, and desmopressin was discontinued. His serum osmolality was 338 mOsm/kg, and his urine osmolality was 362 mOsm/kg. However, his serum sodium started rising again, and on day 18, his sodium level was 161 mEq/L. His urine output was 5.8 L/day, and his urine osmolality was 296 mOsm/kg. He was started on desmopressin 1 µg IV BID. The next day, his sodium was 161 mEq/L, his urine output was 3.6 L, serum osmolality was 346 mOsm/kg, and urine osmolality was 419 mOsm/kg. His copeptin level was 48 pmol/L. Desmopressin was increased up to three times daily till sodium was corrected and urine output normalized. His blood sugar was 144-361 mg/dL during this period. There was no hypercalcemia or severe hypokalemia (K < 3) during the hospital stay. Figure 1 shows the trend of serum sodium and urine osmolarity. Desmopressin was then transitioned to the subcutaneous route and was stopped on day 37 of his hospital stay.

## Discussion

A thorough medical history, clinical assessment, laboratory evaluation, and radiological tests (e.g., pituitary MRI) can help to indicate the possible etiology of polyuria-polydipsia (PP) syndrome. However, they are insufficient to reach a definitive diagnosis [4]. After secondary causes such as diabetes, hypercalcemia, and hypokalaemia have been ruled out, 24-hour urine collection should be used to confirm the presence of hypotonic polyuria [1]. The presence of hyponatremia (plasma sodium <135 mmol/L) or low plasma osmolality (<280 mOsm/kg) is highly suspicious for PP, while a high plasma sodium (≥147 mmol/L) and/or plasma osmolality (>300 mOsm/kg) points toward DI. However, most PP patients have sodium and osmolality levels within normal limits, necessitating additional testing [4].

Complete DI is diagnosed by urine osmolality of less than 300 mOsm/kg despite water deprivation. Patients with central DI show an increase in urine osmolality of more than 50% after desmopressin injection. In contrast, patients with nephrogenic DI remain below this urine osmolality threshold. The urine osmolality of patients with partial central DI or PP usually rises to 300-800 mOsm/kg [4]. For many years, the gold standard for differential diagnosis has been the standard water deprivation test proposed by Miller et al. [5]. Due to several limitations, this test has a diagnostic accuracy of only about 70% [6]. Zerbe and Robertson [7] proposed directly measuring AVP in response to hypertonic saline osmotic stimulation in the 1980s. However, it was never used in clinical practice because of technical limitations and the lack of a commercially available reliable assay [6].

Since the introduction of copeptin, a stable and reliable surrogate marker for AVP, the diagnosis of DI has been re-evaluated. Basal copeptin measurement can reliably diagnose nephrogenic DI without stimulation; however, a stimulation (water deprivation) test is needed to differentiate patients with central DI from those with primary polydipsia [4]. In 2015, a prospective study on various conditions associated with polyuria and polydipsia found that a baseline level of copeptin >21.4 pmol/L can differentiate nephrogenic DI from other diagnoses with excellent sensitivity and specificity (100%) without the use of a water deprivation test [4]. Furthermore, after water deprivation, copeptin of >4.9 pmol/L (at sodium levels >147 mmol/L) allowed for 94.0% specificity and 94.4% sensitivity in distinguishing primary polydipsia from complete or partial central DI [4]. Furthermore, a multicenter prospective trial [8] found that copeptin levels of >4.9 pmol/L had a diagnostic accuracy of 96.5% (93.2% sensitivity and 100% specificity) in distinguishing between central DI and primary polydipsia after stimulation with 3% saline (aiming for a sodium level >150 mmol/L) compared to only 76% for the classical water deprivation test [9]. Recently, the same group proposed arginine-stimulated copeptin measurements as an alternative test in the differential diagnosis of DI in a large cohort of adult patients, with optimal accuracy of 93% at a cutoff of 3.8 pmol/L copeptin at 60 minutes (sensitivity 93%, specificity 92%) [10].

In our case, there is a wide range of evidence that points to both central and nephrogenic DI as a diagnosis. The patient had a high urine output, a high serum sodium level, and low urine osmolality, all of which pointed to DI. He was given desmopressin, and his urine output and sodium levels improved, indicating that the treatment was effective and pointing the diagnosis to central DI. Furthermore, his sodium level fluctuated considerably during treatment, suggesting a diagnosis of central DI rather than nephrogenic DI. The majority of cases of nephrogenic DI are caused by side effects from medications, such as lithium, electrolyte imbalances, such as hypercalcemia or hypokalemia, kidney infiltrating lesions, or vascular problems. Our patient had no hypercalcemia, severe hypokalemia, or any of the other conditions listed above. As a result, nephrogenic DI is a less likely scenario [4].

Meanwhile, the patient also had some findings directing the diagnosis toward nephrogenic DI. First, despite receiving a high dose of desmopressin, the patient’s urine osmolality increased only slightly, supporting the diagnosis of nephrogenic DI. In addition, the patient’s copeptin level was above 40 pmol/L, indicating nephrogenic DI. However, in the United States, copeptin is a research use only test that the Food and Drug Administration has not approved because there is no reliable plasma copeptin assay except in research labs [11]. As a result, the diagnosis should not be based solely on the level of serum copeptin.

## Conclusions

The measurement of copeptin level has high diagnostic accuracy to distinguish between central and nephrogenic DI. Copeptin is taken as a marker of the release of vasopressin. Our patient has mixed findings of partial responsiveness to desmopressin as well as the release of vasopressin from the posterior pituitary, suggestive of both central and nephrogenic components of DI. The copeptin level is paradoxical with the patient’s desmopressin responsiveness in the present case, which makes this case unique.
